# Electroconvulsive Therapy and Risk of Dementia—A Nationwide Cohort Study in Taiwan

**DOI:** 10.3389/fpsyt.2018.00397

**Published:** 2018-09-07

**Authors:** Ching-Wen Chu, Wu-Chien Chien, Chi-Hsiang Chung, Pei-Chun Chao, Hsin-An Chang, Yu-Chen Kao, Yu-Ching Chou, Nian-Sheng Tzeng

**Affiliations:** ^1^Department of Psychiatry, Tri-Service General Hospital, School of Medicine, National Defense Medical Center, Taipei, Taiwan; ^2^Department of Medical Research, Tri-Service General Hospital, National Defense Medical Center, Taipei, Taiwan; ^3^School of Public Health, National Defense Medical Center, Taipei, Taiwan; ^4^Institute of Life Sciences, National Defense Medical Center, Taipei, Taiwan; ^5^Taiwanese Injury Prevention and Safety Promotion Association, Taipei, Taiwan; ^6^Student Counseling Center, National Defense Medical Center, Taipei, Taiwan

**Keywords:** electroconvulsive therapy, cohort study, National Health Insurance Research Database, dementia, risk

## Abstract

**Background:** Electroconvulsive therapy (ECT) is an effective treatment for schizophrenia, bipolar disorder, and major depressive disorder, and a temporary memory loss may occur after ECT. However, the association between ECT in patients with schizophrenia, bipolar disorder, and major depressive disorder, and the risk of dementia is yet to be examined.

**Objective:** This study aimed to clarify as to whether ECT is associated with the risk of dementia after ECT in patients with schizophrenia, bipolar disorder, and major depressive disorder, using Taiwan's National Health Insurance Research Database (NHIRD).

**Methods:** A total of 3,796 enrolled participants (schizophrenia, 46.68%; bipolar disorder, 11.77%; and major depressive disorder, 41.55%) with 994 patients who had received ECT and 2,982 controls matched for sex and age, between January 1, and December 31, 2000, were selected from the NHIRD. After adjusting for confounding factors, Fine and Gray's survival analysis was used to compare the risk of developing dementia during the 10 years of follow-up.

**Results:** Of the study patients, 45 (4.53%) of them developed dementia when compared to 149 (5.0%) in the control group. Fine and Gray's survival analysis revealed that the study patients were not associated with an increased risk of dementia [hazard ratio (HR) = 0.612, 95% confidence interval (CI) = 0.438–1.854, *P* = 0.325]. After adjusting for sex, age, monthly income, urbanization level, geographic region, and comorbidities, the adjusted HR was 0.633 (95% *CI* = 0.448 – 1.895, *P* = 0.304).

**Conclusion:** This study supports that ECT was not associated with the increased risk of dementia in patients with schizophrenia, bipolar disorder, and major depressive disorder, using the NHIRD.

## Introduction

Electroconvulsive therapy (ECT) is an effective treatment for major mental illnesses. ECT is relatively safe and with absolutely no medical contraindications (1, 2). The adverse events and complications are also concerns for the ECT. Moreover, a few complications have been reported in patients undergoing ECT, for example, prolonged seizures (3) and fractures (4). Low death rates were also noted in the ECT (5–8). A short period of memory loss was one of the remarkable complications, which could last as long as several months after the ECT (9–11).

Dementia is a growing global health problem (12–14), which results in a burden for the patient, caregivers, and society (15–17). Since the short period of memory loss, as aforementioned, and amnesia could be the risk factors of subsequent cognitive disorders, including dementia (2, 18–21), it is therefore necessary to examine as to whether ECT is associated with an increase in subsequent dementia.

One Danish cohort study reported that the ECT is not associated with the risk of dementia in patients with affective disorders (22). However, further study is crucial to clarify as to whether there is an association between the ECT in patients with schizophrenia, bipolar disorder, major depressive disorders, and dementia. We therefore aimed to study any incidence of the risk of dementia, prolonged seizures, bone fractures, and death rates from ECT, utilizing the National Health Insurance Research Database (NHIRD) in Taiwan.

## Materials and methods

### Data sources

In this study, we used data from the NHIRD to investigate the association between the patients who have received ECT and developed dementia over a 10-year follow-up period, from the outpatient and hospitalization Longitudinal Health Insurance Database (LHID) (2000–2010). In 1995, the National Health Insurance (NHI) Program was launched, which had contracts with 97% of the medical providers, and enrolled more than 99% of the 23 million population as of June, 2009 (23). The NHIRD includes comprehensive data on the inpatient care, ambulatory care, dental care, and prescription drugs availed by the insurees, as well as their sex and date of birth. Pursuant to the Personal Information Protection Act, individual identifiers are encrypted before release for research. The diagnoses recorded in the NHIRD are coded according to the International Classification of Disease, Ninth Revision, Clinical Modification (ICD-9-CM) (24). The details of the program have been documented in the previous studies (16, 20, 25–29).

All diagnoses of dementia were made by board-certified psychiatrists or neurologists, and for the patients who received ECT, we focused on schizophrenia, bipolar disorders, and major depressive disorders, and all these diagnoses were made by board-certified psychiatrists. The NHI Administration randomly reviews the records of the ambulatory care visits and the inpatient claims, to verify the accuracy of the diagnoses (30). Several studies have demonstrated the accuracy and validity of the diagnoses in the NHIRD (31–33). Therefore, it is suitable to use the NHIRD as the correct tool to complete this study. The ethical committee, Institutional Review Board of the Tri-Service General Hospital, approved this study (TSGH IRB No. 1-104-05-145).

### Study design and sampled participants

**Figure 1:**
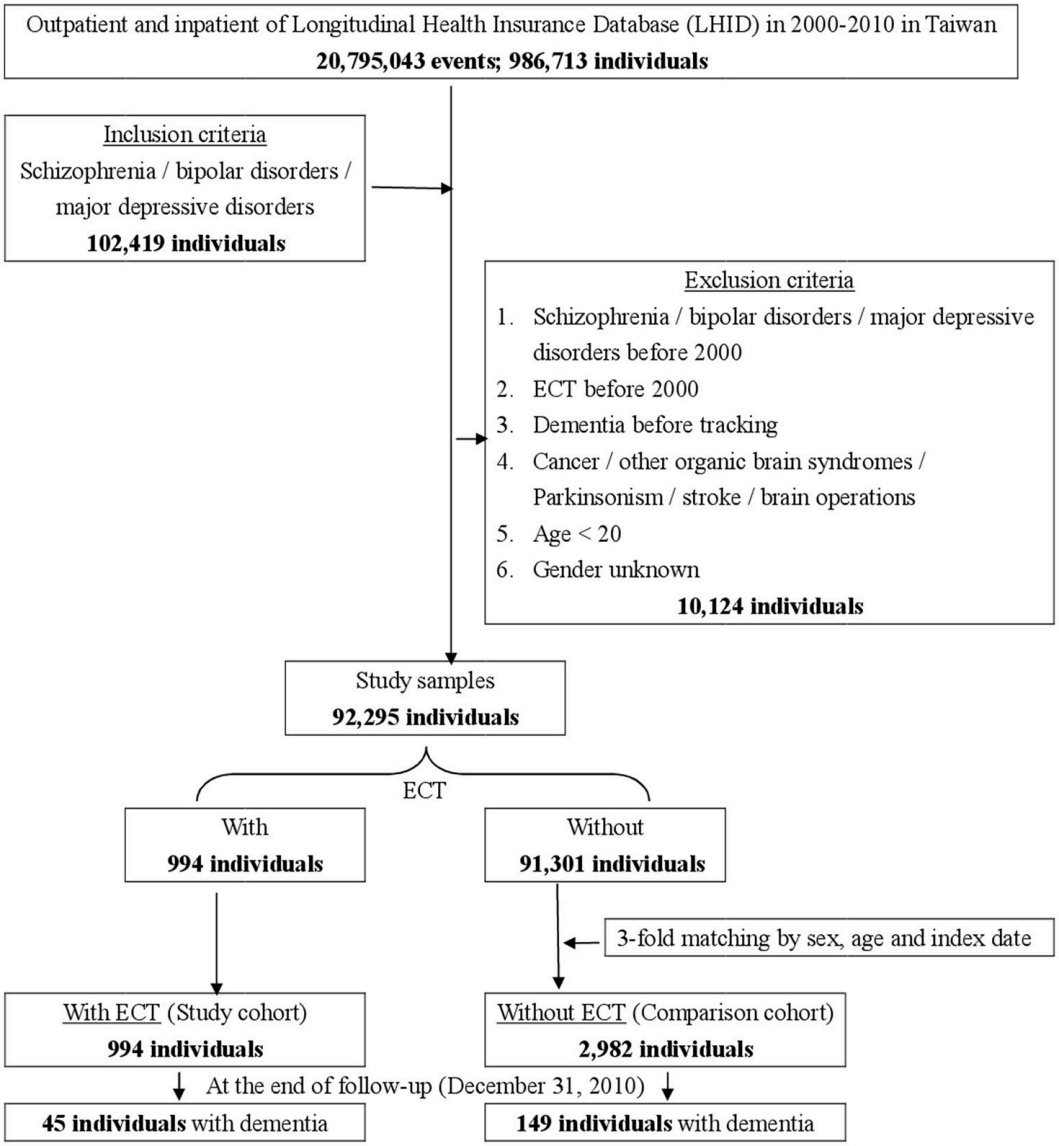
The flowchart of study sample selection from National Health Insurance Research Database in Taiwan.

This study was of a retrospective, matched-cohort design. Patients with schizophrenia, bipolar disorders, or major depressive disorders, who had received ECT, were selected from January 1, to December 31, 2000, according to ICD-9-CM codes: ICD-9-CM 314. In addition, each enrolled patient was required to have made at least three outpatient visits within the 1-year study period for dementia, according to these ICD-9-CM codes. The patients diagnosed with schizophrenia, bipolar disorders, or major depressive disorders before 2000, and the patients who had received ECT before 2000, were excluded. Patients with cancer (ICD-9-CM codes: 140-208), other organic brain syndromes (ICD-9-CM codes: 294), Parkinsonism (ICD-9-CM codes: 332), stroke (ICD-9-CM codes: 430-434), and a history of brain surgeries (ICD-9-CM codes: OP01-OP04) were also excluded. In addition, all the patients aged < 20 years were also excluded. A total of 3,796 enrolled participants (schizophrenia, 46.68%; bipolar disorder, 11.77%; and major depressive disorders, 41.55%), with 994 patients who had received ECT, and 2,982 controls (1:3) matched for sex and age, between January 1, and December 31, 2000, were selected from the NHIRD (Figure 1).

### Covariates

The covariates included sex, age groups (those aged 20–64 and ≧65 years), geographical area of residence (north, center, south, and east of Taiwan), urbanization level of residence (levels 1–4), and insurance premiums (in New Taiwan Dollars (NT$); < 18,000, 18,000–34,999, ≥35,000). The urbanization level of residence was defined according to the population and various indicators of the level of development. Level 1 was defined as a population of >1,250,000, and a specific designation as political, economic, cultural, and metropolitan development. Level 2 was defined as a population between 500,000 and 1,249,999, and as playing an important role in the political system, economy, and culture. Urbanization levels 3 and 4 were defined as a population between 149,999 and 499,999, and < 149,999, respectively (34). The Charlson comorbidity index was used to adjust the factors of the comorbidities (35). Post-ECT prolonged seizures, in-hospital delirium, major cardiovascular adverse events (MACE's), including, acute myocardial infarction (AMI), acute stroke, coronary artery disease (CAD), dysrhythmia, cardiac shock, and deaths within the hospital stay in which the ECT was used, were recorded.

### Major outcome

Data on all of the study participants were collected for the time period beginning January 1, to December 31, 2000, until the onset of dementia (ICD-9-CM codes: 290.0, 290.10, 290.11, 290.12, 290.13, 290.20, 290.21, 290.3, 290.41, 290.42, 290.43, 290.8, 290.9, and 331.0), withdrawal from the NHI program, or the end of 2010. The dementia could be denoted as Alzheimer-type dementia (AD, 290.0x−290.3x, and 331.0), vascular dementia (VaD, 290.4), and other dementias (290.9). In this study, cases of dementia were defined by the criteria of Diagnostic and Statistical Manual of Mental Disorders, Fourth Edition, Text Revision (DSM-IV-TR), and recorded by the ICD-9-CM codes as aforementioned. These cases stand for a significant impairment in social or occupational functioning, and represent a significant decline from a previous level of functioning by memory impairment, and one or more of the following cognitive disturbances such as aphasia, apraxia, agnosia and disturbance in executive functioning. The types of AD, VaD, and other dementia are also defined by the DSM-IV-TR (36).

### Statistical analyses

For the baseline, and the end of the follow-up of the demographic characteristics of the study population, we applied the Chi-square and Fisher exact test for the categorical variability, and a *t*-test for the continual variability. For the survival analysis of the factors of dementia, we used the Kaplan-Meier analysis for the cumulative risk of the follow-up. For each of the dementia analysis factors, the Fine and Gray's competing risk model with both crude hazard ratio (HR) and adjusted HR was applied. Each significant factor and subgroup of dementia type were stratified by using either the Cox regression or Fine and Gray's competing risk model, along with the crude and adjusted HR which were also included for further analysis. All the tests were two-sided, and *p* < 0.05 was considered statistically significant. All the statistical analyses were performed using the IBM SPSS Statistics for Windows, Version 22.0 (IBM Corp., Armonk, NY, USA).

## Results

### Baseline characteristics of the study population

Table 1 shows the sex, age, psychiatric disorder groups, CCI, urbanization and area of residence, and the insurance premiums of the study patients and controls. There were no differences between the study patients and the control groups in sex, age, psychiatric disorder groups, CCI, and insurance premiums. When compared to the non-ECT controls, the ECT cohort tended to live in higher urbanized regions, and in the northern and central regions of Taiwan (*p* < 0.001).

**Table 1:** Characteristics of study at the baseline.

**ECT**	**With**		**Without**		***P***

**variables**	***n***	%	***n***	%	
Total	994	25.00	2,982	75.00	
Study sample subgroup					0.999
Schizophrenia	464	46.68	1,392	46.68	
Bipolar disorders	117	11.77	351	11.77	
Major depressive disorders	413	41.55	1,239	41.55	
Gender					0.999
Male	380	38.23	1,140	38.23	
Female	614	61.77	1,842	61.77	
Age (years)	39.65 ± 12.76		40.40 ± 13.30		0.506
Age group (years)					0.999
20–64	960	96.58	2,880	96.58	
≧65	34	3.42	102	3.42	
CCI_R	0.13 ± 0.29		0.18 ± 0.55		0.643
Urbanization level					< 0.001
1 (The highest)	493	49.60	894	29.98	
2	418	42.05	1,294	43.39	
3	51	5.13	307	10.30	
4 (The lowest)	32	3.22	487	16.33	
Location					< 0.001
Northern Taiwan	430	43.26	1,217	40.81	
Middle Taiwan	218	21.93	754	25.29	
Southern Taiwan	336	33.80	727	24.38	
Eastern Taiwan	9	0.91	264	8.85	
Outlets islands	1	0.10	20	0.67	
Insured premium (NT$)					0.860
< 18,000	984	98.99	2,947	98.83	
18,000–34,999	9	0.91	30	1.01	
≧35,000	1	0.10	5	0.17	

*P, Chi-square/Fisher exact test on category variables and t-test on continue variables; CCI, Charlson Comorbidity Index; NT$, New Taiwan Dollars*.

Supplementary Table 1 depicts the comparison of the three diagnoses— schizophrenia, bipolar disorder, and major depressive disorder. There were more female patients in the groups of bipolar disorder and major depressive disorder. There were also marginal significant differences in the urbanization levels and residence areas in Taiwan among these three diagnostic groups.

### Kaplan-meier model for the cumulative risk of dementia

**Figure 2:**
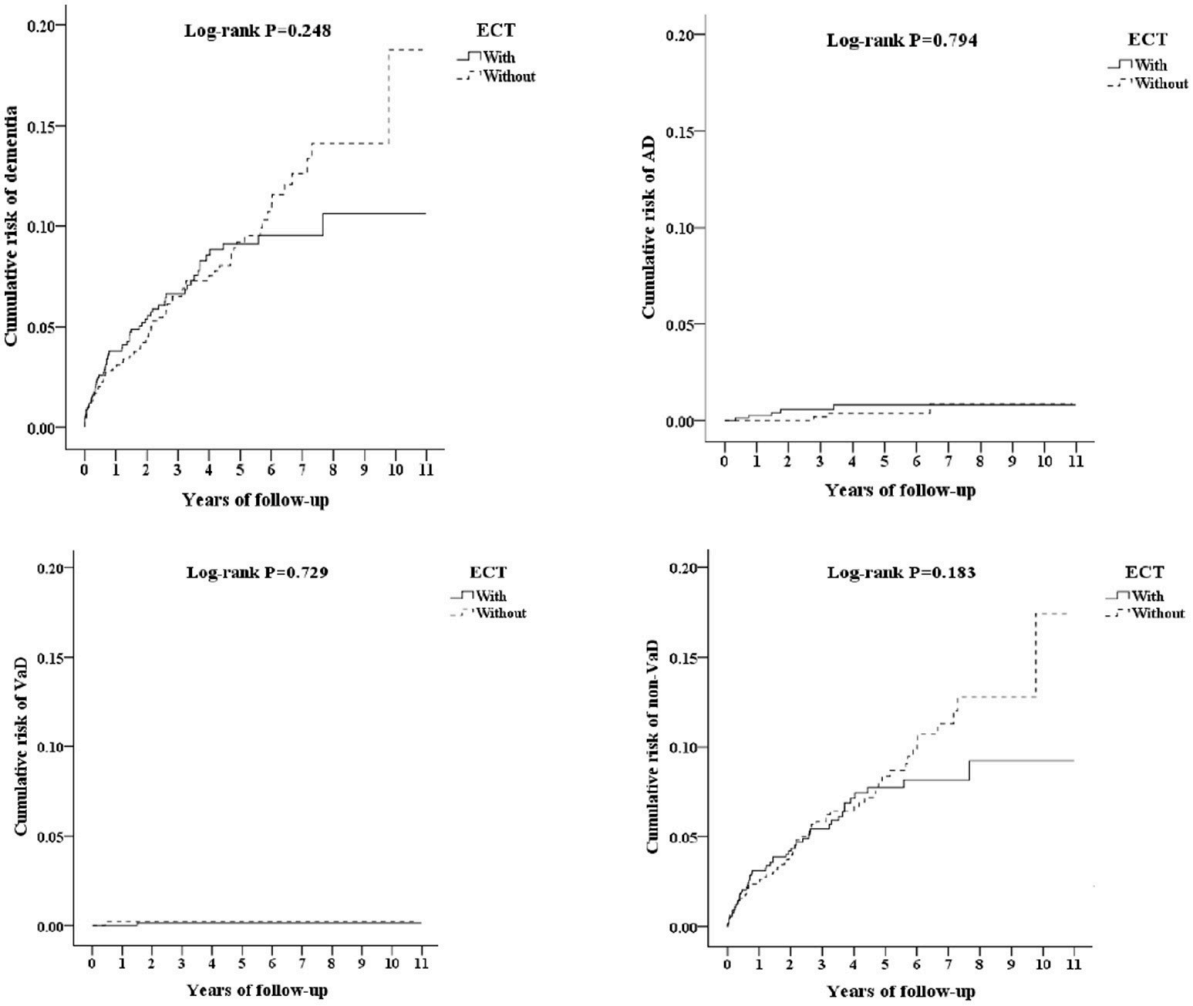
Kaplan-Meier for cumulative risk of dementia among schizophrenia/bipolar disorder/major depressive disorder aged 20 and over stratified by ECT with log-rank test (Left-top, All dementia; Right-top, AD; Left-bottom, VaD; Right-bottom, other dementia).

Figure 2 depicts the Kaplan–Meier analysis for the cumulative incidence of dementia in the study and control groups, and there were no differences between the two groups as being statistically significant, in overall AD, VaD, and other dementia.

### Comparisons of the dementia, ECT-related complications at the end of study, and the readmission rates after ECT-related hospitalizations

Table 2 shows that at the end of the follow-up, 45 out of 994 patients in the study group (4.53%), and 149 out of 2,982 (5.00%) patients in the control group developed dementia. There were only marginal differences in prolonged seizures and in-hospital delirium between the ECT and non-ECT cohorts. In this 10-year follow-up period, none of the study subjects or the control group died.

**Table 2:** Characteristics of study at the end of follow-up.

**ECT**	**With**		**Without**		***P***

**variables**	***n***	%	***n***	%	
Total	994	25.00	2,982	75.00	
Dementia					0.308
Without	949	95.47	2,833	95.00	
With	45	4.53	149	5.00	
Subgroups of dementia					0.695
AD	4	0.40	7	0.23	
VaD	1	0.10	3	0.10	
Other dementia	40	4.02	139	4.66	
Study sample subgroup					0.999
Schizophrenia	464	46.68	1,392	46.68	
Bipolar disorders	117	11.77	351	11.77	
Major depressive disorders	413	41.55	1,239	41.55	
Gender					0.999
Male	380	38.23	1,140	38.23	
Female	614	61.77	1,842	61.77	
Age (years)	46.63 ± 12.76		44.81 ± 13.42		0.127
Age group (years)					0.167
20–64	946	95.17	2,812	94.30	
≧65	48	4.83	170	5.70	
CCI_R	0.17 ± 0.78		0.34 ± 1.18		< 0.001
Spine fractures	0	0	9	0.30	0.075
Non-spine fractures	23	2.31	76	2.55	0.391
Prolonged seizures	7	0.70	1	0.03	< 0.001
Post-ECT delirium/confusions	2	0.20	35	1.17	0.004
MACEs					
AMI	8	0.80	28	0.94	0.847
Acute stroke	37	3.72	123	4.12	0.641
CAD	59	5.94	203	6.81	0.376
Dysrhythmias	82	8.25	242	8.12	0.517
Cardiac shock	3	0.30	6	0.20	0.699
ICD	3	0.30	4	0.13	0.377
SCD	4	0.40	8	0.27	0.509

*P, Chi-square/Fisher exact test on category variables and t-test on continue variables; AD, Alzheimer dementia, VaD, vascular dementia; CCI, Charlson Comorbidity Index; CAD, Coronary artery disease; ICD, Ischemic heart disease; SCD, Sudden cardiac death*.

Supplementary Table 2 depicts the hospital re-admission rates. In these three diagnostic groups, the overall re-admission rates and rates of re-admission for ECT were more than 80 and 20%, respectively.

### Hazard ratios analysis of dementia in the patients who received ECT

Table 3 shows the results of Fine and Gray's survival analysis of the factors associated with the risk of developing dementia. Fine and Gray's survival analysis revealed that the ECT cohort were not more likely to develop dementia, and after adjusting for sex, age, monthly income, urbanization level, geographic region, and comorbidities, the adjusted HR was 0.992 (95% *CI* = 0.896–1.099, *p* = 0.882). For the male patients older than 65 years, and the ECT cohort with prolonged seizures, in-hospital delirium, and acute stroke, were associated with an increased risk of developing dementia that was higher, as in 2.063-, 2.268-, 5.260-, 5.623-, and 2.086-fold (all *p* < 0.001). There were no increased risks of overall dementia and individual types of dementia in the diagnostic subgroups of schizophrenia, bipolar disorder, and major depressive disorder (Table 4).

**Table 3:** Factors of dementia by using Fine and Gray's competing risk model.

**Variables**	**Competing risk in the model**							

	**Crude HR**	**95% CI**	**95% CI**	***P***	**Adjusted HR**	**95% CI**	**95% CI**	***P***
ECT *(reference: without)*	0.612	0.438	1.854	0.325	0.764	0.538	1.086	0.133
Male *(reference: female)*	1.948	1.469	2.584	< 0.001	2.063	1.545	2.756	< 0.001
≧65 years *(reference: 20–64 years)*	2.667	1.797	3.958	< 0.001	2.268	1.476	3.484	< 0.001
CCI_R	1.100	1.027	1.177	0.006	0.992	0.896	1.099	0.882
Spine fractures *(reference: without)*	0	–	–	0.671	0	–	–	0.963
Non-spine fractures *(reference: without)*	0.150	0.021	1.071	0.058	0.120	0.017	0.862	0.035
Prolonged seizures *(reference: without)*	4.310	1.070	17.365	0.040	5.260	1.277	21.666	0.022
Post-ECT delirium/confusions *(reference: without)*	7.063	4.099	12.721	< 0.001	5.623	3.195	9.898	< 0.001
AMI *(reference: without)*	2.589	1.148	5.839	< 0.001	2.203	0.878	5.525	0.092
Acute stroke *(reference: without)*	2.530	1.704	3.757	< 0.001	2.086	1.371	3.175	0.001
CAD *(reference: without)*	1.598	1.076	2.373	0.020	0.971	0.609	1.549	0.903
Dysrhythmias *(reference: without)*	0.834	0.442	1.572	0.574	0.726	0.382	1.380	0.329
Cardiac shock *(reference: without)*	1.591	0.223	11.362	0.643	0.325	0.043	2.451	0.276
ICD *(reference: without)*	0	–	–	0.646	0	–	–	0.963
SCD *(reference: without)*	8.754	2.793	27.436	< 0.001	4.733	1.456	15.380	0.010

*HR, hazard ratio; CI, confidence interval; Adjusted HR: Adjusted for gender, age, comorbidities, urbanization level, location, and insured premium; CCI, Charlson Comorbidity Index; CAD, Coronary artery disease; ICD, Ischemic heart disease; SCD, Sudden cardiac death*.

**Table 4:** Factors of dementia subgroup stratified by study sample by using Cox regression and Fine and Gray's competing risk model.

**ECT** ***(With vs. Without)***		**No competing risk in the model**				**Competing risk in the model**			

**Psychiatric disorders**	**Dementia types**	**Adjusted HR**	**95% CI**	**95% CI**	***P***	**Adjusted HR**	**95% CI**	**95% CI**	***P***
Total	Total	0.777	0.582	1.298	0.236	0.764	0.538	1.086	0.133
	AD	1.496	0.644	3.054	0.374	1.458	0.608	2.460	0.337
	VaD	0.984	0.602	5.723	0.470	0.938	0.692	4.473	0.363
	Other dementia	0.735	0.540	1.276	0.157	0.724	0.501	1.069	0.109
Schizophrenia	Total	0.528	0.316	1.203	0.308	0.518	0.304	1.023	0.152
	AD	-	-	-	-	-	-	-	-
	VaD	0	-	-	0.513	0	-	-	0.423
	Other dementia	0.508	0.293	1.182	0.107	0.499	0.282	1.005	0.096
Bipolar disorders	Total	1.692	0.605	2.861	0.267	1.616	0.584	2.385	0.228
	AD	1.082	0.069	10.193	0.509	1.079	0.065	8.742	0.414
	VaD	-	-	-	-	-	-	-	-
	Other dementia	1.865	0.580	3.460	0.249	1.820	0.563	2.919	0.208
Major depressive disorders	Total	0.942	0.604	3.460	0.249	0.865	0.563	2.919	0.208
	AD	0.956	0.190	2.921	0.433	0.935	0.175	2.362	0.335
	VaD	1.406	0.127	9.442	0.469	1.224	0.104	6.809	0.434
	Other dementia	0.902	0.559	1.072	0.182	0.809	0.527	1.069	0.124

*AD, Alzheimer dementia; VaD, vascular dementia; HR, hazard ratio; CI, confidence interval; Adjusted HR, Adjusted for gender, age, comorbidities, urbanization level, location, and insured premium*.

### Sensitivity analysis for the risk of dementia after ECT in fine and gray's survival analysis model

We have also analyzed the risk of dementia after ECT in the ECT cohort, within the first 3 years, between 3 and 6 years, and more than 6 years, with the ECT cohort not being associated with the increased risk of dementia (Table 5).

**Table 5:** Sensitivity analysis for the risk of dementia after ECT in Fine and Gray's survival analysis model.

**Tracking interval**	**Study sample of subgroups**	**No competing risk in the model**			**Competing risk in the model**		

		**Adjusted HR**	**95% CI**	***P***	**Adjusted HR**	**95% CI**	***P***
Whole period	Total	0.777	0.582–1.298	0.236	0.764	0.538–1.086	0.133
	Schizophrenia	0.528	0.316–1.203	0.308	0.518	0.304–1.023	0.152
	Bipolar disorder	1.692	0.605–2.861	0.267	1.616	0.584–2.385	0.228
	Major depressive disorder	0.942	0.604–3.460	0.249	0.865	0.563–2.919	0.208
0–1 year	Total	0.991	0.651–1.508	0.264	0.971	0.638–1.478	0.213
	Schizophrenia	0.673	0.353–1.398	0.297	0.660	0.346–1.370	0.277
	Bipolar disorder	2.158	0.677–3.324	0.342	2.115	0.663–3.238	0.322
	Major depressive disorder	1.201	0.676–4.020	0.254	1.177	0.662–3.940	0.224
3–6 years	Total	1.821	0.728–4.556	0.200	1.782	0.714–4.469	0.198
	Schizophrenia	1.237	0.398–4.224	0.135	1.211	0.390–4.414	0.112
	Bipolar disorder	7.635	0.837–23.627	0.264	7.490	0.821–23.178	0.245
	Major depressive disorder	2.207	0.756–12.148	0.346	2.165	0.742–11.187	0.313
≧6 years	Total	0.685	0.513–2.065	0.123	0.671	0.502–2.024	0.114
	Schizophrenia	0.462	0.278–1.060	0.377	0.452	0.272–1.038	0.376
	Bipolar disorder	1.491	0.533–2.521	0.277	1.460	0.522–2.486	0.273
	Major depressive disorder	0.803	0.532–3.048	0.298	0.813	0.521–2.984	0.288

*HR, hazard ratio; CI, confidence interval; Adjusted HR: Adjusted for gender, age, comorbidities, urbanization level, location, and insured premium*.

## Discussion

In this cohort study, ECT was not associated with the risk of dementia, in the patients' group with schizophrenia, bipolar disorder, and major depressive disorder, or these psychiatric disorders as a whole. This study echoes the findings of the previous study that the ECT is not associated with an increased risk of dementia in the patients with affective disorders (22), and depicted that neither is the ECT associated with the increased risk of dementia in the patients with schizophrenia, for the first time. The observation was made that the ECT cohort, within the first 3 years, between 3 and 6 years, and more than 6 years, was not associated with the increased risk of dementia, after conducting a sensitivity analysis that also supported this finding.

The patients who received ECT may display short-term cognitive impairment deficits in the processing speed, executive function, and memory (2, 9, 10). Two previous studies examined the risk of dementia in older patients with depression following ECT, and the findings showed that the ECT was associated with an increased risk of dementia; however, these two studies enrolled relatively small sample sizes (*N* = 81, and *N* = 47, respectively) (37, 38). In our study, the risk of dementia in patients did not increase with schizophrenia, bipolar disorder, and major depressive disorders following ECT. One Danish cohort study found that there was no association between ECT and the risk in patients with affective disorders (22). However, in our study, we not only examined the association between patients with affective disorders but also schizophrenia, and the risk of dementia following ECT.

In this study, we did not find any deaths from ECT, in comparison to one previous 17-year nationwide population-based retrospective study in Taiwan, which showed that the in-hospital death rate of patients who received ECT was 0.19% (8). Other studies presented that ECT-related deaths were either very low or none at all (5–7). The ECT cohort with prolonged seizures (39, 40), post-ECT in-hospital delirium (41), and MACE's (9), were complications of ECT. Our study found that these prolonged seizures in-hospital, post-ECT in-hospital delirium, and acute stroke were associated with an increased risk of dementia for the ECT cohort. This could serve as a reminder to the psychiatrists and/or other physicians to screen the risk of these complications before ECT, and be watchful when following-up the ECT patients that have experienced these complications. In addition, older patients and male patients who have received ECT also need careful follow-up.

The ECT has been shown to stimulate neurogenesis, giving rise to the hypothesis that this generation of new cells mediates some of their therapeutic effect (42). The ECT could also induce neuroplasticity, and even increase the volumes in the hippocampus, amygdala, and the regions with prominent connections to the ventromedial prefrontal cortex, and other limbic structures in patients with major depressive disorder (43). For treatment-resistant schizophrenia, the ECT significantly increased the vascular endothelial growth factor, thus, leading to stimulating the neurogenesis in the brain (44). In animal models, the ECT could also provide the neurogenetic effects not only at the hippocampus but also at the frontal brain areas (45). Generally, ECT had a neurogenetic ability and this could be the reason for these patients to preserve their cognitive function after the ECT.

### Strengths of this study

There are several strengths of this study: First, the diagnoses were based on the ICD-9 codes, and several previous studies have demonstrated the accuracy and validity of some diagnoses in the NHIRD, including diabetes mellitus (46, 47), cancer (33, 46, 48), myocardial infarction (46, 49, 50), Tourette syndrome (32), stroke (31, 46, 51, 52), pneumonia (53), sleep apnea (53), pulmonary tuberculosis (54, 55), chronic obstructive pulmonary disease (56), and asthma (56). In addition, the outcomes (46), mortality (46, 51), or comorbidity (46, 51) were also valid in the NHIRD. Some other studies even depicted the concordance between Taiwan's National Health Survey, another health databank, and the NHIRD on a variety of diagnoses (57), medication usage (57), and health system utilizations (57, 58).

Furthermore, the cumulative incidence rates of dementia were 4.53% in the ECT cohort, and 5.0% in the control group, which were close to a previous survey of the nationwide prevalence of dementia in Taiwan (59). Therefore, we propose that this selected population should be considered as a representative for this type of study. Correspondingly, the long-term observation period from 2000 to 2010 allowed for more credibility.

## Limitations

Several limitations should be considered in this study. First, the longest course of our study was followed for only 10 years, and the dementia was a very late onset disease, with the risk being accumulated more in the geriatric patients. This study, therefore, cannot represent the dementia risk in the elderly patients. Second, like many previous NHIRD based studies, they are retrospective and dependent upon the ICD-9-CM codes instead of a direct medical record or the interview data. Therefore, lack of detailed records and misdiagnosis related errors may well occur. Third, this national insurance database cannot provide detailed information that includes the severity of the disease as well as the minor complications. Fourth, in this study, there were three different psychiatric diagnoses groups, therefore we have limitations in the eliminations of the impacts of different diagnoses. Even though we have conducted the analysis of baseline characteristics of the patients with schizophrenia, bipolar disorder, and major depressive disorder (Supplementary Table 1), and the overall or ECT-related readmission rates (Supplementary Table 2), the limitations still exist. However, in the sensitivity test (Table 5), we could also find that the difference of diagnoses with ECT showed no significant impacts on the results, based on the observation that each diagnosis was not associated with the risk of dementia at different time period (0–1, 3–6, ≧6 years) after the ECT. Finally, even though only newly diagnosed dementia would be included in the follow-up period, a protopathic bias, in which the initiation of an exposure occurs in response to an undiagnosed disease (outcome) under study (60), should also be considered since some of the participants in the ECT cohort suffered from cognitive decline before their suicide attempts. However, we have done a sensitivity test as shown in Table 5, which also showed no association between the risk of dementia and ECT.

## Conclusion

There is strong evidence to support that the ECT was not associated with the risk of dementia for patients with schizophrenia, bipolar disorders, and major depressive disorders. Our study also found that the ECT cohort with prolonged seizures in-hospital, post-ECT in-hospital delirium, and acute stroke were associated with the increased risk of dementia for the ECT cohort. This could serve as a reminder to the psychiatrists and/or other physicians to screen the risk of these complications before ECT, and be watchful in following-up the ECT patients that have experienced these complications.

## Author contributions

C-WC, W-CC, and N-ST conceived, designed, and conducted this study. W-CC, C-HC, and P-CC conducted data collection, statistical analysis and interpretation. H-AC, Y-CK, and Y-CC contributed in the data interpretation, C-WC wrote the manuscript. All authors approved this manuscript.

### Conflict of interest statement

The authors declare that the research was conducted in the absence of any commercial or financial relationships that could be construed as a potential conflict of interest.
