# Supplementary material for: Electroconvulsive Therapy and Risk of Dementia—A Nationwide Cohort Study in Taiwan
Source: Front Psychiatry. 2018 Sep 7;9:397. doi: 10.3389/fpsyt.2018.00397 (PMC6138057; doi:10.3389/fpsyt.2018.00397)
Supplement: Supplementary file 1 [file Table_1.DOC]

| **Supplementary Table 1. Comparison of different psychiatric diagnoses that received ECT in this study** | | | | |
| --- | --- | --- | --- | --- |
|  | **Schizophrenia**  **(n=464)** | **Bipolar disorder**  **(n=117)** | **Major depressive disorder**  **(n=413)** | ***P*** |
| **Gender** |  |  |  | <0.001 |
| Male | 231 (49.78%) | 47 (40.17%) | 102 (24.70%) |  |
| Female | 233 (50.22%) | 70 (59.83%) | 311 (75.30%) |  |
| **Age (years)** | 34.43±11.59 | 38.33±13.00 | 41.08±13.06 | <0.001 |
| **Age group (years)** |  |  |  | 0.054 |
| 20-64 | 455 (98.06%) | 111 (94.87%) | 394 (95.40%) |  |
| ≧65 | 9 (1.94%) | 6 (5.13%) | 19 (4.60%) |  |
| **CCI_R** | 0.04±0.22 | 0.08±0.30 | 0.09±0.34 | 0.022 |
| **Urbanization level** |  |  |  | 0.007 |
| 1 (The highest) | 205 (44.18%) | 58(49.57%) | 230 (55.69%) |  |
| 2 | 206 (44.39%) | 52 (44.44%) | 160 (38.74%) |  |
| 3 | 32 (6.90%) | 5 (4.27%) | 14 (3.39%) |  |
| 4 (The lowest) | 21 (4.53%) | 2 (1.72%) | 9 (2.18%) |  |
| **Location** |  |  |  | 0.003 |
| Northern Taiwan | 187 (40.30%) | 54 (46.15%) | 189 (45.76%) |  |
| Middle Taiwan | 129 (27.80%) | 25 (21.37%) | 64 (15.50%) |  |
| Southern Taiwan | 145 (31.25%) | 37 (31.62%) | 154 (37.29%) |  |
| Eastern Taiwan | 2 (0.43%) | 1 (0.86%) | 6 (1.45%) |  |
| Outlets islands | 1 (0.22%) | 0 (0%) | 0 (0%) |  |
| **Insured premium (NT$)** |  |  |  | 0.618 |
| <18,000 | 459 (98.92%) | 117 (100%) | 408 (99.79%) |  |
| 18,000-34,999 | 5 (1.08%) | 0 (0%) | 4 (0.97%) |  |
| ≧35,000 | 0 (0%) | 0 (0%) | 1 (0.24%) |  |

***P*: Chi-square / Fisher exact test on category variables and one-way ANOVA on continue variables; CCI: Charlson Comorbidity Index; NT$: New Taiwan Dollars; ECT**: **electroconvulsive therapy**
