# Supplementary material for: Electroconvulsive Therapy and Risk of Dementia—A Nationwide Cohort Study in Taiwan
Source: Front Psychiatry. 2018 Sep 7;9:397. doi: 10.3389/fpsyt.2018.00397 (PMC6138057; doi:10.3389/fpsyt.2018.00397)
Supplement: Supplementary file 2 [file Table_2.DOC]

| **Supplementary Table 2. Hospital re-admission rates after the ECT-related hospitalization** | | |
| --- | --- | --- |
|  | Overall Re-admission rates (%) | ECT Re-admission rates (%) |
| **Schizophrenia (n=464)** | 86.21 | 27.37 |
| **Bipolar disorder (n=117)** | 81.20 | 25.64 |
| **Major depressive disorder (n=413)** | 84.75 | 23.00 |

**ECT:** electroconvulsive therapy
